# Association between low back movement control impairment test results and low back pain – Northern Finland birth cohort study 1966

**DOI:** 10.1186/s12891-026-10176-6

**Published:** 2026-07-11

**Authors:** Peetu Robert Rytkönen, Jani Takatalo, Hannu Luomajoki, Petteri Oura, Heidi Ruotsalainen, Marika Tuiskunen, Kari Niemi, Jaro Karppinen

**Affiliations:** 1 Research Unit of Health Sciences and Technology, University of Oulu, Oulu, Finland; 2 Loisto Terveys, Oulu, Finland; 3 Institute of Physiotherapy, School of Health Professions, Zurich University of Applied Sciences, Winterthur, Switzerland; 4 Medical Research Center Oulu, Oulu University Hospital, University of Oulu, Oulu, Finland; 5 Oulu University of Applied Sciences, Oulu, Finland; 6 OMT Fysioterapia Niemi, Salo, Finland; 7 Terveystalo Pulssi, Turku, Finland; 8 Wellbeing Services County of South Karelia, Lappeenranta, Finland

**Keywords:** Movement control impairment, Low back pain, Sciatica

## Abstract

**Background:**

Movement control impairment (MCI) tests of patients with chronic low back pain (LBP) have shown good validity. However, the association between MCI test results and LBP has not been evaluated in a population-based cohort.

**Methods:**

The participants of this cross-sectional study consisted of 1907 members of the Northern Finland Birth Cohort 1966. Their assessment included the MCI tests of sitting knee extension (SKE), prone knee bend (PKB), one leg stance (OLS), rocking backwards (RB) and waiter’s bow (WB). The tests were classified as positive or negative. The SKE, PKB and OLS tests were performed on the left and right sides separately and then summed up to include both sides. Each MCI test evaluates at least one movement direction; flexion (SKE, RB, WB), extension (PKB), rotation (SKE, PKB, OLS), flexion-rotation (SKE, OLS) and extension-rotation (PKB, OLS). The participants’ pain level was categorized as mild, moderate, or severe according to the frequency and perceived bothersomeness of LBP. The outcomes were LBP, sciatica and prolonged sciatica (duration over three months). Multinomial regression analysis was used to analyse the association between each MCI direction and the symptoms, and adjusted for body mass index, sex, socioeconomic status, psychological symptoms, physical activity, and smoking. The reference categories were mild symptoms and negative MCI test results of the respective direction.

**Results:**

Three positive left-side rotation MCI test results were associated with moderate (odds ratio [OR] 2.81; 95% confidence interval (CI) 1.22–6.50) and severe (2.98; 1.20–7.40) LBP. Two and three positive rotation tests (both sides) were also associated with severe LBP (2.31; 1.02–5.24 and 3.03; 1.27–7.24, respectively). Two positive left-side flexion-rotation test results were associated with moderate LBP (1.83; 1.01–3.29) and one positive flexion-rotation test result (both sides) with severe LBP (1.84; 1.08–3.10). One positive right-side flexion-rotation test result was negatively associated with moderate prolonged sciatica (0.28; 0.10–0.77).

**Conclusions:**

Positive rotational MCI test results were associated with moderate and severe LBP in this large-scale population-based cohort. The reason for the association between positive rotational test results and LBP, especially left-sided, requires further research.

**Supplementary Information:**

The online version contains supplementary material available at https://doi.org/10.1186/s12891-026-10176-6.

## Background

 Low back pain (LBP) is a common symptom globally [1]. It is the leading cause of global years lived with disability, although age-standardized rates have decreased modestly over the past three decades [1, 2]. Non-specific LBP, with no pathoanatomical source of pain [3], is the most prevalent type of LBP (85–90%) [3–5]. LBP is a complex condition and has multiple contributors to pain and disability, including biological, psychological and social factors [1]. A recent study found that the spreading of pain, irrespective of its location, can be predicted using a common psychosocial framework in which the major risk factors are mood, sleep, neuroticism (i.e., feeling fed-up), life stressors and a body mass index (BMI) of over 30 [6]. Poor general health, which includes smoking, is also a recognized risk factor for LBP and sciatica [7]. The pathophysiological mechanisms of LBP are largely unknown, whereas in the case of sciatica, inflammation of the lumbar nerve roots is considered to be a key mechanism [8].

Movement control impairment (MCI) syndromes have been suggested as a possible cause of non-specific LBP [9]. MCI means that movement control of the lower back is poor during functional activities [5]. The purpose of MCI exercises is to improve the physical and cognitive function of the impaired movement direction. Motor control exercises differ from MCI exercises as they focus more on activating isolated deep trunk muscles in complex static, dynamic and functional tasks [10]. Motor control training involves synergy between the deep muscles and the superficial global muscles [11]. MCI exercises also involve this synergy of muscle activation, but they focus more on developing controlled movement in different directions than on activating specific deep muscles in movement variations.

Of the MCI tests, the waiter’s bow (WB), pelvic tilt (PT), one leg stance (OLS), sitting knee extension, (SKE), rocking backward (RB) (quadruped position in which pelvis moves forwards and backwards) and prone knee bend (PKB) tests have shown good validity among patients with chronic LBP [12, 13]. However, there is also evidence that only PKB and OLS have good reliability and therefore clinicians prefer these tests [14]. It has been suggested that MCI test results are more often positive among patients with LBP than among asymptomatic individuals [13]. Ramalingam et al. [15] have reported similar results, but not with the PT test, which is difficult to perform. It seems that rotational MCI tests in particular are more difficult to perform on LBP patients than on asymptomatic people [15, 16].

Only a few studies have evaluated the association between MCI test results and LBP. Furthermore, no population-based studies have been conducted, even though they could help us understand these associations. Our study hypothesis was that positive MCI test results are associated with more intense LBP and sciatica, and positive MCI test results may be more rotation directed.

## Methods

### Study sample

**Fig. 1:**
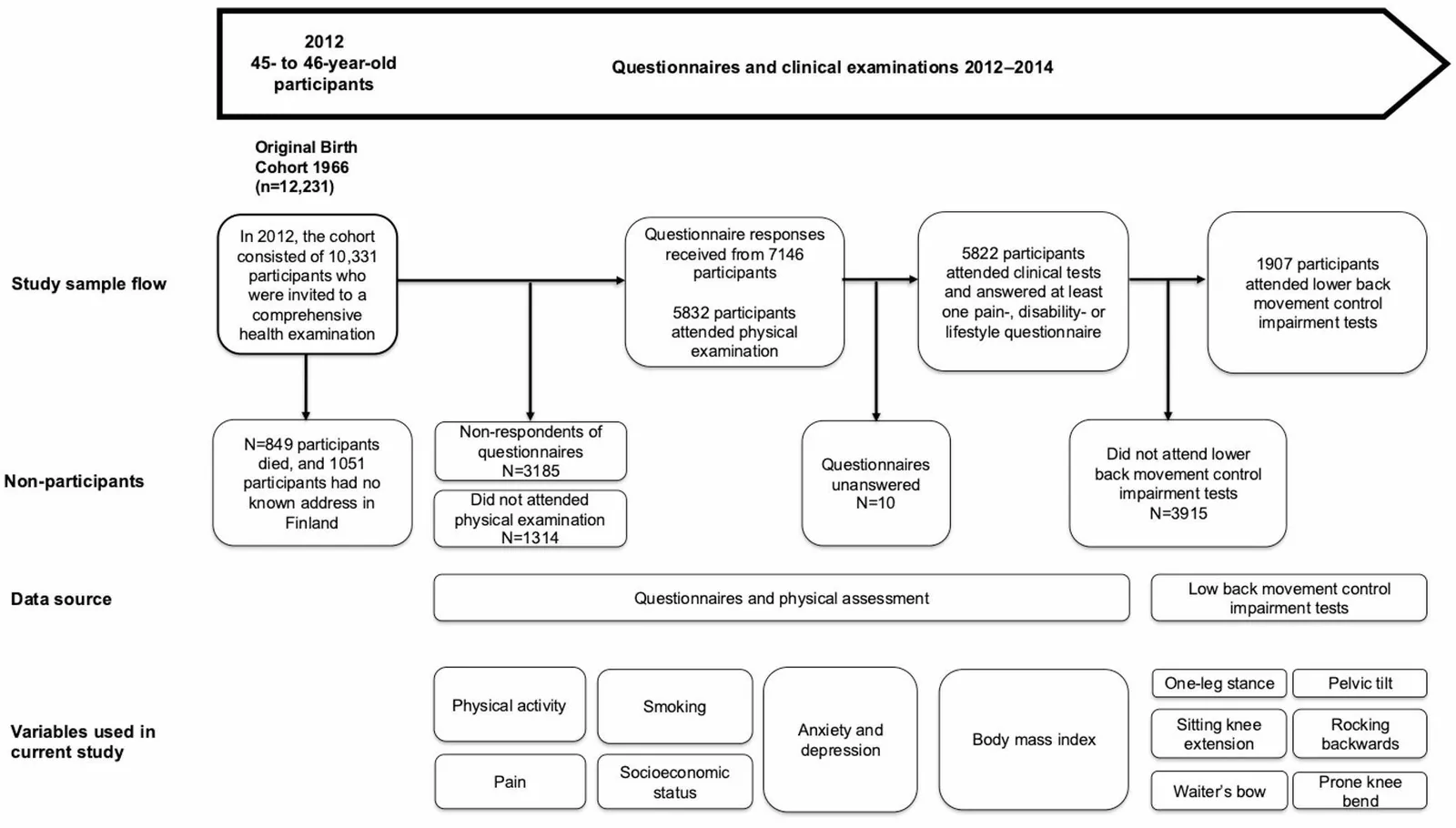
Study flow chart

Figure 1 presents the study flow chart. The study was reviewed by the Ethical Committee of the Northern Ostrobothnia Hospital District, Oulu, Finland (§ 94/2011). The participants of this cross-sectional study were drawn from the Northern Finland Birth Cohort 1966 (NFBC1966). Originally, the NFBC1966 consisted of 12,231 members [17]. In 2012, 84.5% of the NFBC1966 members (*N* = 10,331) were invited to a 46-year follow-up health examination, which started at the beginning of the same year and ended on 1 March 2014. 69% of the 46-year follow-up participants (*N* = 7146) answered questionnaires on pain, disability and lifestyle factors, and 57% (*N* = 5832) attended a one-day physical assessment, which included BMI measurement. Almost everyone who attended the physical assessment also answered at least one questionnaire (*N* = 5822) [18]. Finally, 33% (*N* = 1907) of the one-day physical assessment participants who responded to at least one of the questionnaires participated in the MCI tests. Participation to the 46-year follow-up health examination was voluntary for all NFBC1966 members. Background, lifestyle, and health survey is one of the four questionnaires in NFBC1966 46-year-follow-up [18]. From this survey LBP, sciatica and confounders were formed to our study.

### MCI tests

The participants were instructed in each MCI test by a physiotherapist, and their performance was videorecorded. Three physiotherapists were involved in this phase of the study. Altogether, the participants underwent six MCI tests: the SKE, PKB, OLS, RB, WB and the PT. The SKE was filmed from the back, the OLS from the front and the PKB, RB, WB and PT from the right side.

The PT test was excluded because it can test many different MCIs, for example, sagittal plane MCI in both the thoracic and lumbar spine, which would make its interpretation more complicated than those of the other five MCI tests. All the tests were analysed from the recorded video after the assessment and were categorized as negative, positive or undecided for the specified testing direction. If it was not possible to evaluate the test from the videorecording, MCI was categorized as undecided, and therefore, excluded from the analysis. The reliability of assessing the MCI tests from a video recording was determined by two physiotherapists. The first physiotherapist was a musculoskeletal physiotherapist who had > 10 years of experience and was specialized in MCI rehabilitation. For the intra-rater reliability of the MCI tests, the first physiotherapist evaluated the same MCI tests again at least one month after the first evaluation. The second physiotherapist was also a musculoskeletal physiotherapist – an Orthopaedic Manual Physical Therapist (OMPT), with > 30 years of experience and a Kinetic Control Accredited Tutor (KCAT), i.e. was certified to teach MCI. For the inter-rater reliability of the MCI tests, the results of these two physiotherapists were compared.

The tests were categorized by their MCI test directions: flexion (WB, SKE, RB), rotation (OLS, SKE, PKB), flexion-rotation (OLS, SKE), extension-rotation (OLS, PKB) and extension (PKB). The rotation, flexion-rotation, and extension-rotation MCI tests were more specifically categorized by their test side (left, right) and the sum score of the both-side tests (both). Altogether, there were 11 different test directions: flexion, rotation (right), rotation (left), rotation (both), flexion-rotation (right), flexion-rotation (left), flexion-rotation (both), extension-rotation (right), extension-rotation (left), extension-rotation (both) and extension. Finally, the MCI sum score was calculated by adding up SKE (both, if either side was positive, the test was marked as positive), PKB (both), OLS (both), WB and RB as all test directions. The results were then categorized into three groups: 0, 1–2 and 3–5 positive tests, on the basis of earlier findings [13].

### LBP and sciatica

**Fig. 2:**
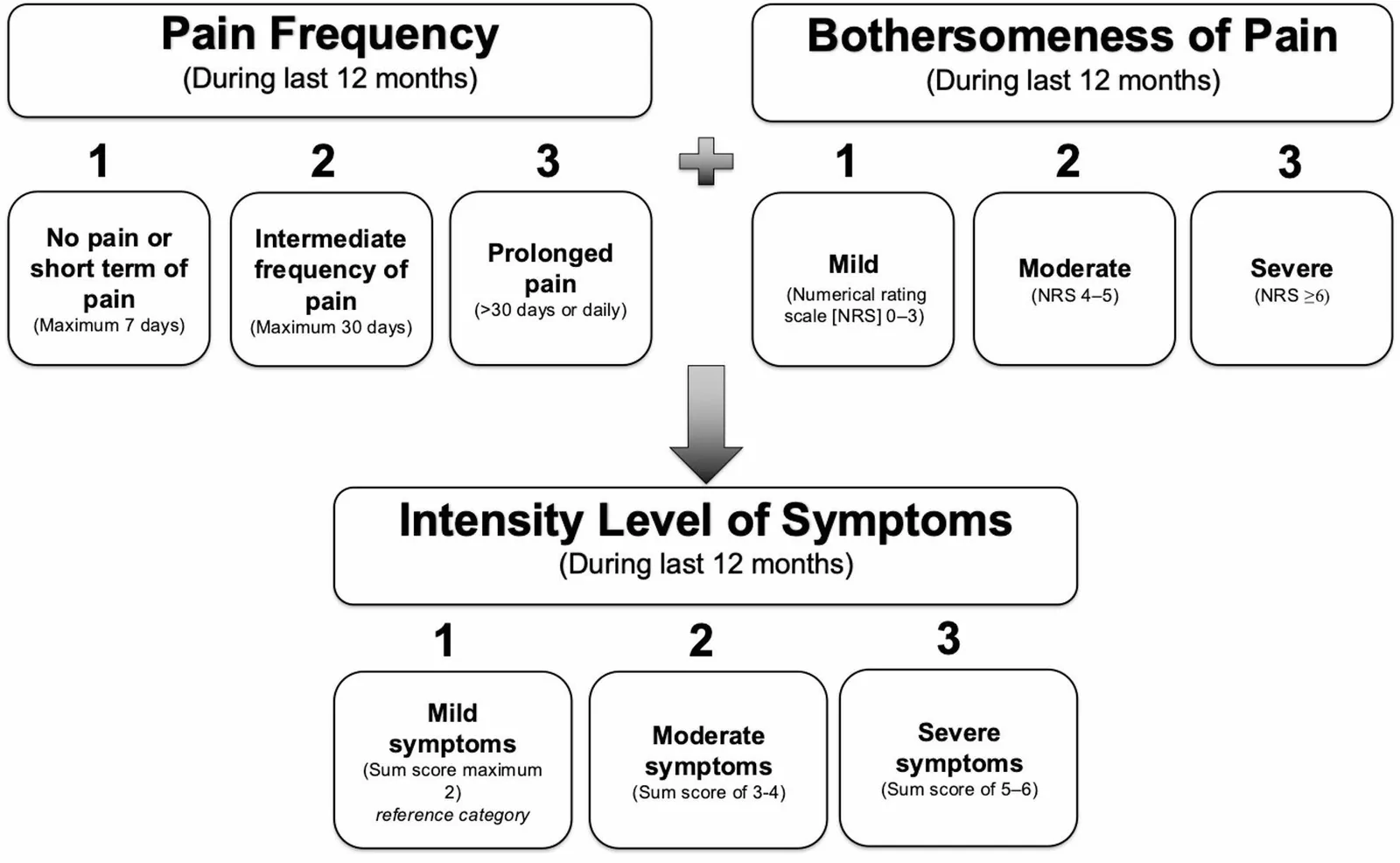
Derivation of the primary pain variable

The questions used to evaluate the frequency of pain were: (1) Have you had aches or pains in your lower back during the last 12 months? (2) Have you had aches or pains in your lower back with radiating pain or numbness below the knee level in the last 12 months? and (3) Have you had aches or pains in your lower back that have lasted over three months with radiating pain or numbness below the knee level in the last 12 months? The response options for the frequency of pain were ‘none’ (0), ‘1 to 7 days’ (1), ‘8 to 30 days’ (2), ‘more than 30 days but not daily’ (3) and ‘daily’ (4). The responses were categorized as: 1) no pain or short-term pain (at most 7 days), 2) intermediate frequency of pain (at most 30 days) and 3) prolonged pain (over 30 days or daily). For prolonged pain frequency, a similar definition has been applied earlier [19]. When the participants reported any pain, they were asked to estimate its overall bothersomeness on a numerical rating scale (NRS) ranging from 0 to 10, in which 0 equalled ‘no pain’ and 10 equalled ‘total disability’. The level of bothersomeness was classified as 1) mild (NRS 0–3), 2) moderate (NRS 4–5), and 3) severe (NRS ≥ 6). Figure 2 shows how the sum score of the pain frequency and bothersomeness classifications were calculated for each pain category (LBP, sciatica and prolonged sciatica) and then re-categorized into three groups: mild symptoms (maximum sum score of 2, reference), moderate symptoms (sum score of 3–4), and severe symptoms (sum score of 5–6). These three groups formed this study’s main pain variable for LBP, sciatica and prolonged sciatica.

### Confounders

The socioeconomic status of the participants was determined by asking “What is your professional education?” the responses to which were classified into three groups: (1) no professional education or professional class (= low level), (2) other education, uncompleted education, vocational education, institutional school (= moderate level), and (3) university of applied sciences and university (= high level).

Level of physical activity was determined by asking “How often do you exercise so that you are out of breath?” and physical activity was classified into three groups: (1) less than once a week (= inactive), (2) 1–3 times a week (= moderately active), and (3) at least 4 times a week (= active).

Smoking status was determined by asking two questions: (1) “Have you ever smoked in your life?” and (2) “Do you currently smoke?”. Smoking was classified into three groups: (1) never smokers i.e. non-smokers, (2) former smokers and (3) current smokers.

Finally, the participants’ depression and anxiety were evaluated using the Hopkins Symptom Checklist-25 questionnaire (HSCL-25), which contains 25 difficulties and symptoms that the participants have experienced during the last week: (1) suddenly scared for no reason, (2) feeling fearful, (3) faintness, dizziness, or weakness, (4) nervousness or shakiness inside, (5) heart pounding or racing, (6) trembling (tremors), (7) feeling tense or keyed up, (8) headaches, (9) spells of terror or panic, (10) feeling restless, can’t sit still, 11) feeling low in energy–slowed down, 12) blaming yourself for things, 13) crying easily, 14) loss of sexual interest or pleasure, 15) poor appetite, 16) difficulty falling asleep, staying asleep, 17) feeling hopeless about the future, 18) feeling blue, 19) feeling lonely, 20) feeling trapped or caught, 21) worrying too much about things, 22) feeling no interest in things, 23) thoughts of ending your life, 24) feeling everything is an effort and 25) feelings of worthlessness. Each item was scored 1 (not at all), 2 (some), 3 (quite a bit), or 4 (very much) and the sum of these scores was divided by 25 to obtain the final HSCL-25 score. In this study, the HSCL-25 was dichotomized: no psychological symptoms (HSCL-25 score between 1.0 and 1.54) and significant psychological symptoms (HSCL-25 score between 1.55 and 4.0) [20].

### Statistical analysis

The differences between the MCI participants (MCI population) and the remaining cohort members (non-participant cohort members), as well as the differences between the sexes were analysed using the Chi square test or the T-test in the case of BMI. The intra- and inter-rater reliability of the MCI tests was proven in a subsample using kappa statistics and classified into six categories (< 0.00 poor agreement, 0.01–0.20 slight agreement, 0.21–0.40 fair agreement, 0.41–0.60 moderate agreement, 0.61–0.80 substantial agreement and 0.81–1.00 almost perfect agreement) [21, 22]. From a clinical standpoint, confidence in findings may be reduced by kappa values below 0.60, whereas ≥ 0.80 is recommended for robust interpretation [23]. Multinomial regression analysis was used to analyse the association of each MCI direction independently and the sum score of all MCI test directions (explanatory variables) with LBP, sciatica and prolonged sciatica (outcomes). The analysis was adjusted for BMI, sex, socioeconomic status, psychological symptoms, physical activity and smoking. The reference categories were mild symptoms of pain and negative findings in all the MCI tests. The cut-off p-value to reject the null hypothesis was 0.05, and the analyses were performed using IBM SPSS^®^ Statistics software, version 29 (Chicago, IL, USA).

## Results

### Characteristics of MCI population

The MCI population consisted of 1907 participants (53.7% females). Table 1 shows the demographic and pain-related characteristics of the MCI population. There were significant differences between sexes in BMI (*p* = 0.003), sciatica symptoms (*p* = 0.003), socioeconomic status (*p* = 0.015), smoking (*p* < 0.001) and psychological status (*p* = 0.004). The LBP group had a higher percentage of participants with severe symptoms (29.6%) than the sciatica (15.0%) or prolonged sciatica (5.5%) groups (Table 1). LBP frequency was higher (*p* = 0.023) in the MCI population than among the non-participants, but otherwise the participants and non-participants did not differ (Table 2). All the MCI tests were more likely to be positive among females (*p* < 0.001; Supplementary file 1).

**Table 1:** Characteristics of study population (MCI population) by sex

	Both sexes	Female	Male	Sex difference
	(n=1907)	(n=1024, 53.7%)	(n=883, 46.3%)	(p-value)^•^
BMI, n (%)	1906 (99.9%)	1023 (53.6%)	883 (46.3%)	
Mean (SD)	26.9 (4.9)	26.6 (5.3)	27.2 (4.2)	0.003
Missing data	1 (0.1%)	1 (0.1%)	0	
LBP^*^, n (%)	1465 (76.8%)	802 (78.4%)	663 (75.1%)	
Mild symptoms	581 (39.7%)	306 (38.2%)	275 (41.5%)	0.360
Moderate symptoms	450 (30.7%)	248 (30.9%)	202 (30.5%)	
Severe symptoms	434 (29.6%)	248 (30.9%)	186 (28.0%)	
Missing data	442 (23.2%)	221 (21.6%)	220 (24.9%)	
Sciatica^*^, n (%)	1620 (85.0%)	876 (85.6%)	744 (84.3%)	
Mild symptoms	1141 (70.4%)	587 (67.0%)	554 (74.5%)	0.003
Moderate symptoms	237 (14.6%)	137 (15.6%)	100 (13.4%)	
Severe symptoms	242 (15.0%)	152 (17.4%)	90 (12.1%)	
Missing data	287 (15.0%)	147 (14.4%)	139 (15.7%)	
Prolonged sciatica^*^, n (%)	1702 (89.3%)	919 (89.8%)	783 (88.7%)	
Mild symptoms	1561 (91.7%)	837 (91.1%)	724 (92.4%)	0.585
Moderate symptoms	48 (2.8%)	28 (3.0%)	20 (2.6%)	
Severe symptoms	93 (5.5%)	54 (5.9%)	39 (5.0%)	
Missing data	205 (10.7%)	104 (10.2%)	100 (11.3%)	
Socioeconomic status^#^, n (%)	1907 (100.0%)	1024 (100.0%)	883 (100.0%)	
Low level	110 (5.8%)	47 (4.6%)	63 (4.1%)	0.015
Moderate level	1216 (69.0%)	700 (68.4%)	616 (69.8%)	
High level	481 (25.2%)	277 (27.1%)	204 (23.1%)	
Missing data	0	0	0	
Smoking^##^, n (%)	1710 (89.7%)	919 (89.7%)	791 (89.6%)	
Non-smokers	805 (42.2%)	480 (46.9%)	325 (36.8%)	<0.001
Not currently smokers	605 (31.7%)	288 (28.1%)	317 (35.9%)	
Currently smokers	300 (15.7%)	151 (14.7%)	149 (16.9%)	
Missing data	197 (10.3%)	105 (10.3%)	92 (10.4%)	
Physical activity^###^, n (%)	1825 (95.7%)	983 (96.00%)	842 (95.4%)	
Inactive	476 (25.0%)	241 (23.5%)	235 (26.6%)	0.258
Moderately active	1050 (55.1%)	578 (56.4%)	472 (53.5%)	
Well active	299 (15.7%)	164 (16.0%)	135 (15.3%)	
Missing data	82 (4.3%)	41 (4.0%)	41 (4.6%)	
Psychological status^####^, n (%)	1742 (91.3%)	928 (90.6%)	814 (92.2%)	
No psychological symptoms	1395 (73.2%)	719 (70.2%)	676 (76.6%)	0.004
Psychological symptoms	347 (18.2%)	209 (20.4%)	138 (15.6%)	
Missing data	165 (8.7%)	96 (9.4%)	69 (7.8%)	

^*^LBP, sciatica and prolonged sciatica symptoms (lasting >3 months) are defined separately symptoms related to intensity level of pain, over 12 months, which is the sum score of frequency and bothersomeness of pain. The sum score is categorized as: mild symptoms 2, moderate symptoms 3–4 and severe symptoms 5–6

^#^Socioeconomic status, defined by level of education

^##^Smoking, defined by smoking

^###^Physical activity, defined by weekly amount of brisk exercise

^####^Psychological status, defined by HSCL-25

**Table 2:** Non-participants of remaining cohort compared to participants of movement control impairment (MCI) tests

	Non-participants	MCI population	Statistical significance
	(n=5238, 73.3%)	(n=1907, 26.7%)	(p-value)^°°^
Sex			
Female	2823 (53.9%)	1024 (53.7%)	0.882
Male	2415 (46.1%)	883 (46.3%)	
BMI	3908 (74.6%)	1906 (99.9%)	
Mean (SD^°^)	26.9 (4.9)	26.9 (4.9)	0.837
Missing data	1330 (25.4%)	1 (0.1%)	
LBP frequency^*^	4497 (85.9%)	1661 (87.1%)	
No pain or short-term pain	2485 (55.3%)	878 (52.9%)	0.023
Intermediate pain	990 (22.0%)	350 (21.1%)	
Prolonged pain	1022 (22.7%)	433 (26.0%)	
Missing data	741 (14.1%)	246 (12.9%)	
Sciatica frequency^*^	4833 (92.3%)	1819 (95.4%)	
No pain or short-term pain	3888 (80.4%)	1418 (78.0%)	0.073
Intermediate pain	454 (9.4%)	197 (10.8%)	
Prolonged pain	491 (10.2%)	204 (11.2%)	
Missing data	405 (7.7%)	88 (4.6%)	
Prolonged sciatica frequency^*^	4839 (92.4%)	1812 (95.0%)	
No pain or short-term pain	4450 (92.0%)	1682 (92.8%)	0.504
Intermediate pain	83 (1.7%)	28 (1.6%)	
Prolonged pain	306 (6.3%)	102 (5.6%)	
Missing data	399 (7.6%)	95 (5.0%)	
Bothersomeness of LBP^§^	4065 (77.6%)	1499 (78.6%)	
Mild disability	2326 (57.2%)	845 (56.4%)	0.755
Moderate disability	675 (16.6%)	261 (17.4%)	
High disability	1064 (26.2%)	393 (26.2%)	
Missing data	1173 (22.4%)	408 (21.4%)	
Bothersomeness of sciatica^§^	4419 (84.4%)	1648 (86.4%)	
Mild disability	3465 (78.4%)	1263 (76.6%)	0.138
Moderate disability	333 (7.5%)	120 (7.3%)	
Hight disability	621 (14.1%)	265 (16.1%)	
Missing data	819 (15.6%)	259 (13.6%)	
Bothersomeness of prolonged sciatica^§^	4713 (90.0%)	1735 (91.0%)	
Mild disability	4324 (91.8%)	1605 (92.5%)	0.445
Moderate disability	110 (2.3%)	32 (1.8%)	
High disability	279 (5.9%)	98 (5.7%)	
Missing data	525 (10.0%)	172 (9.0%)	

°SD, Standard deviation

*LBP, sciatica and prolonged sciatica (lasting >3 months) defined separately by frequency of pain over 12 months, and categorized as 1) no pain or short-term pain (no pain or 1–7 days), 2) intermediate pain frequency (8–30 days) and 3) prolonged pain (>30 days or daily pain)

^§^LBP, sciatica and prolonged sciatica (lasting >3 months) defined separately by perceived bothersomeness of pain over 12 months, and categorized as 1) mild disability (0–3), 2) moderate disability (4–5) and 3) high disability (≥6) (0=no bothersomeness at all and 10=worst imaginable bothersomeness)

°°Statistical significance was analysed using the Chi-square test, but for BMI as a continuous variable, the T-test was used

### Reliability of videorecording evaluation

The results concerning the intra- and inter-rater reliability of the MCI tests are presented in Supplementary file 2. The intra- and inter-rater reliability assessment involved 99 participants per test, except for the inter-rater reliability assessment of the RB and PKB tests (*N* = 97 and 96, respectively). Intra-rater reliability was almost perfect for every MCI test. The PKB and all the MCI tests (Kappa 0.62; 95% confidence interval [CI] 0.47–0.78 and 0.63; CI 0.60–0.66, respectively) had excellent inter-rater reliability agreement. The WB, PT, and all the MCI tests without PT (Kappa 0.43; CI 0.25–0.62, 0.49; CI 0.26–0.71 and 0.59; CI 0.54–0.64, respectively) had moderate inter-rater reliability agreement. The SKE, RB and OLS tests (Kappa 0.27; CI 0.12–0.41, 0.27; CI 0.13–0.41 and 0.21; CI 0.03–0.38, respectively) had fair inter-rater reliability agreement.

### Association between MCI and severity of LBP, sciatica and prolonged sciatica

The MCI test results, stratified by pain categories, are shown in Supplementary file 3, and the adjusted multinomial regression analyses are shown in Table 3. Moderate and severe LBP were associated with left-side rotation MCI when all three tests were positive (odds ratio [OR] 2.81; CI 1.22–6.60 and OR 2.98; CI 1.20–7.40, respectively). Severe LBP was associated with both left- and right-side rotation MCI when two and three test results were positive (OR 2.31; CI 1.02–5.24 and OR 3.03; CI 1.27–7.24, respectively). Moderate LBP was associated with left-side flexion-rotation MCI when two test results were positive (OR 1.83, CI 1.01–3.29). Severe LBP was associated with both-side flexion-rotation MCI when one test result was positive (OR 1.84; CI 1.08–3.10). Prolonged sciatica of moderate severity was negatively associated with right-side flexion-rotation MCI with one positive test result (OR 0.28; CI 0.10–0.77). No significant associations were found between positive MCI test results and sciatica in the adjusted model.

**Table 3:** Association between movement control impairment (MCI) tests and low back pain (LBP) and sciatica*

Movement control impairment direction^•^	Number of positive tests	All intensity levels of symptoms refer to pain reported over last 12 months^••^					
		Moderate LBP	Severe LBP	Moderate sciatica	Severe sciatica	Moderate prolonged sciatica	Severe prolonged sciatica
Flexion	1	0.93 (0.50–1.73)^•••^	0.72 (0.38–1.35)	1.91 (0.91–4.01)	1.22 (0.58–2.58)	1.74 (0.32–9.40)	0.77 (0.25–2.39)
	2	1.18 (0.63–2.21)	1.10 (0.58–2.09)	1.18 (0.53–2.63)	0.55 (0.23–1.30)	1.31 (0.23–7.52)	0.21 (0.04–1.09)
	3	1.29 (0.64–2.61)	0.87 (0.41–1.81)	1.93 (0.83–4.51)	1.83 (0.82–4.06)	1.15 (0.15–8.71)	0.94 (0.27–3.26)
Rotation (right)	1	0.84 (0.34–2.12)	0.91 (0.33–2.45)	1.28 (0.41–4.00)	0.80 (0.27–2.37)	0.66 (0.13–3.44)	0.83 (0.17–4.16)
	2	0.97 (0.39–2.39)	1.23 (0.47–3.24)	1.27 (0.41–3.88)	1.07 (0.38–3.04)	0.48 (0.09–2.45)	0.99 (0.21–4.69)
	3	1.58 (0.62–4.04)	1.67 (0.61–4.56)	1.39 (0.44–4.39)	0.96 (0.33–2.82)	0.48 (0.09–2.73)	0.86 (0.17–4.39)
Rotation (left)	1	1.40 (0.62–3.12)	1.86 (0.77–4.47)	0.67 (0.27–1.67)	3.06 (0.68–13.70)	1.08 (0.12–9.68)	1.03 (0.21–5.19)
	2	1.87 (0.86–4.07)	1.94 (0.82–4.58)	1.14 (0.48–2.68)	2.76 (0.62–12.26)	1.23 (0.15–10.44)	1.12 (0.24–5.35)
	3	2.81 (1.22–6.50)	2.98 (1.20–7.40)	1.21 (0.48–3.05)	4.05 (0.89–18.28)	1.55 (0.16–14.64)	2.03 (0.41–10.21)
Rotation (both)	1	1.19 (0.60–2.35)	2.15 (0.95–4.87)	1.61 (0.66–3.90)	1.21 (0.46–3.18)	0.73 (0.13–3.97)	0.49 (0.14–1.80)
	2	1.45 (0.73–2.86)	2.31 (1.02–5.24)	1.95 (0.80–4.74)	1.45 (0.56–3.79)	1.15 (0.23–5.78)	0.92 (0.28–3.11)
	3	1.93 (0.92–4.08)	3.03 (1.27–7.24)	1.46 (0.55–3.87)	1.70 (0.63–4.62)	0.62 (0.09–4.22)	0.80 (0.21–3.09)
Flexion-rotation (right)	1	1.14 (0.66–1.95)	1.18 (0.68–2.06)	0.69 (0.38–1.25)	1.31 (0.68–2.54)	0.28 (0.10–0.77)	1.73 (0.57–5.23)
	2	1.56 (0.88–2.78)	1.34 (0.74–2.43)	0.98 (0.52–1.82)	1.24 (0.62–2.61)	0.38 (0.13–1.12)	1.39 (0.43–4.57)
Flexion-rotation (left)	1	1.13 (0.66–1.93)	1.70 (0.94–3.08)	0.72 (0.39–1.34)	1.58 (0.71–3.52)	0.58 (0.17–1.98)	1.87 (0.54–6.54)
	2	1.83 (1.01–3.29)	1.83 (0.96–3.49)	1.23 (0.64–2.38)	1.99 (0.86–4.60)	0.83 (0.22–3.16)	1.92 (0.51–7.27)
Flexion-rotation (both)	1	1.28 (0.79–2.08)	1.84 (1.08–3.10)	1.05 (0.60–1.84)	1.21 (0.65–2.28)	0.41 (0.14–1.25)	2.00 (0.71–5.62)
	2	1.39 (0.82–2.37)	1.57 (0.88–2.81)	1.14 (0.61–2.12)	1.46 (0.74–2.86)	0.54 (0.16–1.80)	1.11 (0.33–3.72)
Extension-rotation (right)	1	0.71 (0.42–1.20)	1.26 (0.70–2.25)	0.82 (0.46–1.47)	1.30 (0.68–2.47)	1.33 (0.38–4.64)	2.05 (0.70–6.00)
	2	0.83 (0.50–1.40)	1.52 (0.85–2.72)	0.81 (0.45–1.44)	1.30 (0.69–2.47)	0.73 (0.20–2.67)	1.66 (0.56–4.93)
Extension-rotation (left)	1	1.08 (0.68–1.71)	1.15 (0.71–1.87)	1.09 (0.63–1.89)	1.31 (0.74–2.34)	1.75 (0.39–7.92)	0.96 (0.40–2.32)
	2	1.15 (0.73–1.82)	1.34 (0.83–2.15)	1.09 (0.64–1.89)	1.37 (0.78–2.42)	2.35 (0.54–10.35)	1.41 (0.61–3.29)
Extension-rotation (both)	1	0.98 (0.67–1.43)	1.24 (0.83–1.84)	1.29 (0.83–2.02)	1.46 (0.91–2.33)	2.28 (0.77–6.77)	0.95 (0.48–1.91)
	2	1.07 (0.71–1.61)	1.32 (0.86–2.02)	0.96 (0.59–1.58)	1.54 (0.94–2.51)	1.34 (0.40–4.47)	1.36 (0.67–2.77)
Extension	1	1.09 (0.82–1.45)	1.19 (0.89–1.59)	1.10 (0.79–1.54)	1.05 (0.75–1.45)	1.30 (0.64–2.65)	1.05 (0.64–1.73)
All tests	1-2	0.85 (0.23–3.06)	0.93 (0.23–3.76)	0.87 (0.25–2.97)	1.44 (0.17–12.07)	0.51 (0.05–5.13)	0.52 (0.05–4.99)
	3-5	1.43 (0.40–5.16)	1.36 (0.34–5.50)	0.90 (0.26–3.12)	2.12 (0.25–17.68)	0.52 (0.05–5.14)	0.49 (0.05–4.88)

^*^LBP, sciatica and prolonged sciatica categorized as intensity levels of symptoms referr to pain reported over last 12 months (mild, moderate and severe symptoms)

^•^Reference category for movement controls tests was “no positive movement control impairment tests for each direction tested”

^••^Reference category for the reported pain over 12 months was “mild LBP, sciatica and prolonged sciatica symptoms”

^•••^Odds ratio (OR) is outside brackets and 95% confidence interval (CI) inside brackets. OR (and 95% confidence intervals) calculated using multinomial regression analyses, adjusted for BMI, sex, smoking, socioeconomic status

## Discussion

In this population-based cohort study, moderate and severe LBP were associated with rotational MCI. Both-side, but especially left-side MCI was associated with LBP. Generally, two or three positive rotational MCI test results are needed for a significant association with LBP. However, severe LBP was associated with both-side flexion-rotation MCI when only one test result was positive. Prolonged sciatica of moderate severity was negatively associated with right-side flexion-rotation MCI with one positive test. To the authors’ knowledge, the current study was the first population-based study to study the association between MCI and lower back symptoms.

The latest American Physical Therapy Association (APTA) guidelines strongly recommend MCI exercises for chronic LBP patients who have MCI [24]. MCI exercises of the lower back are believed to reduce pain intensity in the short term and disability in both the short and long term more effectively than other studied interventions among patients who have non-specific LBP with MCI [10]. These studies have shown that changing movement strategies might improve functioning and reduce disability among LBP patients. Therefore, in terms of movement control, we believe that choosing the individually most important exercises on the basis of the identification of MCI direction is crucial. However, the association between LBP intensity and MCI remains contradictory. Bauer et al. [25] found an association between pain intensity and the variability of lumbar movement patterns and task-specific actions, but not direction-specific MCI. Future studies should test the validity of this hypothesis.

Significant associations were found between positive MCI test results and LBP but not sciatica or prolonged sciatica. However, moderate and severe symptoms were less frequently reported in the sciatica and prolonged sciatica groups than in the LBP group, which may have reduced the statistical power of these analyses. Moreover, local pain in the lower back may change lumbar movement control, whereas sciatica symptoms may contribute less to lower back movement.

We do not completely understand the reason for the left-side domination in the results. We can speculate that right-handed participants can more precisely control neural activation on the right side than on their weaker left side. However, we did not know the proportions of right- and left-handed participants in our study population, although, in general, right-handed people are known to be more prevalent, at least in Finland [26]. The reason for the negative association between one positive MCI test result in right-side flexion-rotation and prolonged sciatica also remains unclear.

The association between the higher number of positive MCI test results and LBP can be explained by the diminished ability to perceive movement. It should also be noted that combined rotation MCI directions have more tests than the other directions. Ramalingam et al. [15] used the same MCI tests as those used in our study, but added PT, and found that all MCI tests except for PT were more likely to be positive among symptomatic than asymptomatic individuals. As in our study, the rotation-direction MCI test results were more likely to be positive among people with LBP [15]. Previously, Van Dillen et al. [27] have stated that rotation coupled with extension, and rotation and extension alone are the most valid MCI tests. Our study results are in line with those of earlier studies and highlight the difficulty of performing rotation-direction MCI tests among individuals with severe LBP [16]. Previously, Carlsson and Rasmussen-Barr [14] have recommended PKB and OLS in clinical use because of their moderate to good reliability. In our study, PKP had almost perfect intra-rater reliability and substantial inter-rater reliability, so it still can be recommended in terms of reliability. Yet its validity remains unclear because extension or extension-rotation was not associated with LBP in our study. In our study, OLS had almost perfect intra-rater reliability, but only fair inter-rater reliability.

Our study has several strengths. The intra-rater reliability of all the MCI tests was almost perfect, which lends credibility to our results. The inter-rater reliability of our study was fair to moderate, which is slightly lower than that in the previous reliability study [12]. However, our favourable intra- and inter-rater reliability results are the strength of this study. The great number of members in this large-scale population-based cohort is another strength. In addition, the cohort data consisted of many essential questionnaires related to lifestyle and pain, which enabled analyses of the MCI test results and pain variables to be adjusted for the known confounders used in pain research. All the NFBC1966 members participated in our study voluntarily, so selection of participants was not possible. However, the participants of the current study were similar to the non-participants in terms of BMI, sex and all pain variables, except for LBP frequency. Also, the results of the study are credible and are in line with previous evidence, especially in terms of the association between rotation MCI and LBP [15, 16, 27].

Our study also had some limitations. We did not categorize the asymptomatic patients as their own group. We used pain categories that allowed some bothersomeness and frequency of pain during the last 12 months as a reference category. This decision was made because having some LBP-related pain and bothersomeness during the past 12 months is quite normal, as the one-year prevalence of LBP is 38% [28]. In the analysis, we had to combine ’more than 30 days but not daily’ and ‘daily pain’ categories as one group due to lack of power. Unfortunately, category ’more than 30 days but not daily’ does not necessarily describe prolonged pain (> 3 months). Similar pain level classification for musculoskeletal pain has been used previously [29]. Furthermore, quite a high number of participants were lost, as frequency and bothersomeness were combined as one variable. Moreover, several MCI test results were recorded inadequately or performed inaccurately, and therefore, rejected from the final study sample, as this made it impossible to evaluate MCI. Moreover, pain behaviour was not observable in video recordings due to low audio quality. Another weakness of this study is that we had no other information on current pain or its location during the MCI tests. In previous studies, PT has been categorized as extension control test with good overall intra- and inter reliability [12]. We decided to exclude the PT test from our study because of concomitant movement of the lumbar spine during the test, which is contrary to the rest of the MCI test battery. Therefore, the rationale for the PT test is not in line with the other MCI assessments. Moreover, PT can be determined as a thoracic spine MCI. However, excluding PT from the analysis may have affected the association of the extension tests with symptoms and, therefore, it can be seen as a weakness of our study. Although inter-rater reliability was only fair for extension MCI tests (SKE, RB and OLS), intra-rater reliability was almost perfect, which suggests their reliability in clinical use.

Some issues regarding the generalization of our results should be considered. Our study population had higher LBP frequency than the non-participants, but the difference was small, and we believe it is not likely that it affects the generalizability of the results. In our study, 57% of the MCI participants and 56% of the non-participants reported LBP during the last 12 months, whereas in a Finnish national survey, 44% of females and 50% of males had experienced LBP in the last 30 days at the age of 40–49 [30]. The high prevalence of LBP in our study may be explained by symptomatic individuals being more willing to participate. Sex differences in BMI, socioeconomic status, smoking and psychological status are consistent with previous findings; however, further research is required to reach definitive conclusions regarding sex-based patterns in sciatica [31–34]. In our study, males exhibited higher BMIs, which, combined with age, has been found to be associated with positive lower back MCI test findings [35].

Future studies should incorporate data on LBP and self-perceived movement quality during MCI testing, utilizing more individualized protocols tailored to patient symptoms. Furthermore, fear of movement and other cognitive barriers should be accounted for in both future research and clinical practice, as these may adversely influence MCI test performance.

## Conclusion

This large-scale population-based cohort study examined the associations between lower back MCI test results and LBP. Our results show that rotational (rotation or flexion-rotation) MCI is associated with moderate and severe LBP. The association with LBP required two to three positive left-side rotational MCI test results and one to three positive both-side rotational MCI test results. Positive both-side rotational test results were associated with severe LBP. MCI test results were not associated with sciatica. Our findings suggest that focusing on flexion-rotation and rotation MCI tests can provide more nuanced information regarding movement-related LBP.

## Supplementary Information

Supplementary Material 1.

Supplementary Material 2.

Supplementary Material 3.

## Data Availability

NFBC data are available from the University of Oulu, Infrastructure for Population Studies. Permission to use the data for research purposes can be applied for via an electronic material request portal. In the use of data, we follow the EU general data protection regulation (679/2016) and the Finnish Data Protection Act. The use of personal data is based on the cohort participant’s written informed consent in their most recent follow-up study, which may cause limitations to its use. Please contact the NFBC project center (NFBCprojectcenter(at)oulu.fi) and visit the cohort website (www.oulu.fi/nfbc) for more information.
