# Supplementary material for: Association between low back movement control impairment test results and low back pain – Northern Finland birth cohort study 1966
Source: BMC Musculoskelet Disord. 2026 Jul 11;27:842. doi: 10.1186/s12891-026-10176-6 (PMC13647630; doi:10.1186/s12891-026-10176-6)
Supplement: Supplementary file 1 [file 12891_2026_10176_MOESM1_ESM.docx]

| **Supplementary file 1**. Characteristics of study population by movement control impairment test category^§^. | | | | | | | | |
| --- | --- | --- | --- | --- | --- | --- | --- | --- |
|  | | Both sexes | | Female | | Male | | Sex difference |
|  | | (n=1907) | | (n=1024, 53.7%) | | (n=883, 46.3%) | | (p-value)^•^ |
| Flexion^*^ | | 803 (42.1%) |  | 494 (48.2%) |  | 309 (35.0%) |  |  |
|  | 0 positive test results | 155 (19.3%) | | 59 (11.9%) | | 96 (31.1%) | | <0.001 |
|  | 1 positive test result | 256 (31.9%) | | 158 (32.0%) | | 98 (31.7%) | |  |
|  | 2 positive test results | 241 (30.0%) | | 163 (33.0%) | | 78 (25.2%) | |  |
|  | 3 positive test results | 151 (18.8%) | | 114 (23.1%) | | 37 (12.0%) | |  |
|  | Missing data^§§^ | 1104 (57.9%) |  | 530 (51.8%) |  | 574 (65.0%) |  |  |
|  | |  | |  | |  | |  |
| Rotation (left)^**^ | | 1110 (58.2%) |  | 712 (69.5%) |  | 398 (45.1%) |  |  |
|  | 0 positive test results | 60 (5.4%) | | 15 (2.1%) | | 45 (11.3%) | | <0.001 |
|  | 1 positive test result | 285 (25.6%) | | 139 (19.5%) | | 146 (36.6%) | |  |
|  | 2 positive test results | 469 (42.3%) | | 302 (42.4%) | | 167 (42.0%) | |  |
|  | 3 positive test results | 296 (26.7%) | | 256 (36.0%) | | 40 (10.1%) | |  |
|  | Missing data | 797 (41.8%) | | 312 (30.5%) | | 485 (54.9%) | |  |
|  | |  | |  | |  | |  |
| Rotation (right)^***^ | | 1126 (59.0%) |  | 714 (69.7%) |  | 412 (46.7%) |  |  |
|  | 0 positive test results | 48 (4.3%) | | 12 (1.7%) | | 36 (8.7%) | | <0.001 |
|  | 1 positive test result | 261 (23.2%) | | 114 (16.0%) | | 147 (35.7%) | |  |
|  | 2 positive test results | 496 (44.0%) | | 321 (44.9%) | | 175 (42.5%) | |  |
|  | 3 positive test results | 321 (28.5%) | | 267 (37.4%) | | 54 (13.1%) | |  |
|  | Missing data | 781 (41.0%) | | 310 (30.3%) | | 471 (53.3%) | |  |
|  | |  | |  | |  | |  |
| Rotation (both)^****^ | | 1068 (56.0%) |  | 692 (67.6%) |  | 376 (42.6%) |  |  |
|  | 0 positive test results | 85 (8.0%) | | 23 (3.3%) | | 62 (16.5%) | | <0.001 |
|  | 1 positive test result | 308 (28.8%) | | 156 (22.5%) | | 152 (40.4%) | |  |
|  | 2 positive test results | 438 (41.0%) | | 317 (45.8%) | | 121 (32.2%) | |  |
|  | 3 positive test results | 237 (22.2%) | | 196 (28.4%) | | 41 (10.9%) | |  |
|  | Missing data | 839 (44.0%) | | 332 (32.4%) | | 507 (57.4%) | |  |
|  | |  | |  | |  | |  |
| Flexion-rotation (left)^#^ | | 1122 (58.8%) |  | 717 (70.0%) |  | 405 (45.9%) |  |  |
|  | 0 positive test results | 149 (13.3%) | | 54 (7.5%) | | 95 (23.4%) | | <0.001 |
|  | 1 positive test result | 569 (50.7%) | | 327 (45.6%) | | 242 (59.8%) | |  |
|  | 2 positive test results | 404 (36.0%) | | 336 (46.9%) | | 68 (16.8%) | |  |
|  | Missing data | 785 (41.2%) | | 307 (30.0%) | | 478 (54.1%) | |  |
|  | |  | |  | |  | |  |
| Flexion-rotation (right)^##^ | | 1142 (59.9%) |  | 721 (70.4%) |  | 421 (47.7%) |  |  |
|  | 0 positive test results | 151 (13.2%) | | 59 (8.2%) | | 92 (21.8%) | | <0.001 |
|  | 1 positive test result | 573 (50.2%) | | 329 (45.6%) | | 244 (58.0%) | |  |
|  | 2 positive test results | 418 (36.6%) | | 333 (46.2%) | | 85 (20.2%) | |  |
|  | Missing data | 765 (40.1%) | | 303 (29.6%) | | 462 (52.3%) | |  |
|  | |  | |  | |  | |  |
| Flexion-rotation (both)^###^ | | 1081 (56.7%) |  | 698 (68.2%) |  | 383 (43.4%) |  |  |
|  | 0 positive test results | 211 (19.5%) | | 76 (10.9%) | | 135 (35.2%) | | <0.001 |
|  | 1 positive test result | 522 (48.3%) | | 346 (49.6%) | | 176 (46.0%) | |  |
|  | 2 positive test results | 348 (32.2%) | | 276 (39.5%) | | 72 (18.8%) | |  |
|  | Missing data | 826 (43.3%) | | 326 (31.8%) | | 500 (56.6%) | |  |
|  | |  | |  | |  | |  |
| Extension-rotation (left)^####^ | | 1743 (91.4%) |  | 954 (93.2%) |  | 789 (89.4%) |  |  |
|  | 0 positive test results | 184 (10.6%) | | 67 (7.0%) | | 117 (14.8%) | | <0.001 |
|  | 1 positive test result | 684 (39.2%) | | 329 (34.5%) | | 355 (45.0%) | |  |
|  | 2 positive test results | 875 (50.2%) | | 558 (58.5%) | | 317 (40.2%) | |  |
|  | Missing data | 164 (8.6%) | | 70 (6.8%) | | 94 (10.6%) | |  |
|  | |  | |  | |  | |  |
| Extension-rotation (right)^¶^ | | 1720 (90.2%) |  | 942 (92.0%) |  | 778 (88.1%) |  |  |
|  | 0 positive test results | 149 (8.7%) | | 48 (5.1%) | | 101 (13.0%) | | <0.001 |
|  | 1 positive test result | 695 (40.4%) | | 337 (35.8%) | | 358 (46.0%) | |  |
|  | 2 positive test results | 876 (50.9%) | | 557 (59.1%) | | 319 (41.0%) | |  |
|  | Missing data | 187 (9.8%) | | 82 (8.0%) | | 105 (11.9%) | |  |
|  | |  | |  | |  | |  |
| Extension-rotation (both)^¶¶^ | | 1699 (89.1%) |  | 930 (90.8%) |  | 769 (87.1%) |  |  |
|  | 0 positive test results | 326 (19.2%) | | 121 (13.0%) | | 205 (26.7%) | | <0.001 |
|  | 1 positive test result | 783 (46.1%) | | 418 (45.0%) | | 365 (47.4%) | |  |
|  | 2 positive test results | 590 (34.7%) | | 391 (42.0%) | | 199 (25.9%) | |  |
|  | Missing data | 208 (10.9%) | | 94 (9.2%) | | 114 (12.9%) | |  |
|  | |  | |  | |  | |  |
| Extension^¶¶¶^ | | 1883 (98.7%) |  | 1017 (99.3%) |  | 866 (98.1%) |  |  |
|  | 0 positive test results | 747 (39.7%) | | 297 (29.2%) | | 450 (52.0%) | | <0.001 |
|  | 1 positive test result | 1136 (60.3%) | | 720 (70.8%) | | 416 (48.0%) | |  |
|  | Missing data | 24 (1.3%) | | 7 (0.7%) | | 17 (1.9%) | |  |
|  | |  | |  | |  | |  |
| All tests^¶¶¶¶^ | | 735 (38.5%) |  | 466 (45.5%) |  | 269 (30.5%) |  |  |
|  | 0 positive test results | 26 (3.5%) | | 3 (0.6%) | | 23 (8.6%) | | <0.001 |
|  | 1–2 positive test results | 299 (40.7%) | | 172 (36.9%) | | 127 (47.2%) | |  |
|  | 3–5 positive test results | 410 (55.8%) | | 291 (62.5%) | | 119 (44.2%) | |  |
|  | Missing data | 1172 (61.5%) | | 558 (54.5%) | | 614 (69.5%) | |  |
| ^§^Sum score of specific direction tests (0=negative test, 1=positive test) are calculated and results are subcategorized by number of positive results.  ^*^Flexion test includes sitting knee extension (SKE) for both sides separately, waiter’s bow (WB) and rocking backwards (RB).  ^§§^ Undecided results of tests were reported as missing data.  ^**^Rotation (left) test includes SKE (left), prone knee bend (PKB) left, and one leg stance (OLS) left.  ^***^Rotation (right) test includes SKE (right), PKB (right), and OLS (right).  ^****^Rotation (both) test includes SKE (both), PKB (right), and OLS (both).  ^#^Flexion-rotation (left) test includes SKE (left), and OLS (left).  ^##^Flexion-rotation (right) test includes SKE (right), and OLS (right).  ^###^Flexion-rotation (both) test includes SKE (both), and OLS (both).  ^####^Extension-rotation (left) test includes PKB (left), and OLS (left).  ^¶^Extension-rotation (right) test includes PKB (right), and OLS (right).  ^¶¶^Extension-rotation (both) test includes PKB (both), and OLS (both).  ^¶¶¶^Extension test includes PKB (both).  ^¶¶¶¶^All tests category includes SKE (both), PKB (both), OLS (both), WB and RB. Sum score of all five tests were subcategorized to 0, 1–2 and 3–5 positive tests.  ^•^Sex differences in the positive MCI tests were analysed using the Chi-square test. | | | | | | | | |
