# Supplementary material for: Association between low back movement control impairment test results and low back pain – Northern Finland birth cohort study 1966
Source: BMC Musculoskelet Disord. 2026 Jul 11;27:842. doi: 10.1186/s12891-026-10176-6 (PMC13647630; doi:10.1186/s12891-026-10176-6)
Supplement: Supplementary file 2 [file 12891_2026_10176_MOESM2_ESM.docx]

| **Supplementary file 2**. Inter- and intra-rater reliability of movement control impairment (MCI) tests from videorecordings. | | | | | | | |
| --- | --- | --- | --- | --- | --- | --- | --- |
|  | | | | | | | |
|  | | Intra-rater reliability of MCI tests | | | Inter-rater reliability of MCI tests | | |
|  | |  | | |  | | |
|  | | Physiotherapist 1 first evaluation | Physiotherapist 1 second evaluation | Cohen’s Kappa^**^ | Physiotherapist 1 | Physiotherapist 2 | Cohen’s Kappa |
|  | |  |  |  |  |  |  |
| Waiter’s bow | |  |  |  | |  |  |
|  | Negative | 25 | 25 |  | 25 | 44 |  |
|  | Positive | 73 | 73 | 1.00 (N/A) | 73 | 54 | 0.43 (0.25–0.62) |
|  | Undecided | 1 | 1 |  | 1 | 1 |  |
|  | Total N | 99 | 99 |  | 99 | 99 |  |
|  | |  |  |  |  |  |  |
| Single knee extension | |  |  |  | |  |  |
|  | Negative | 27 | 24 |  | 27 | 45 |  |
|  | Positive | 36 | 36 | 0.94 (0.88–1.00) | 36 | 18 | 0.27 (0.12–0.41) |
|  | Undecided | 36 | 39 |  | 36 | 36 |  |
|  | Total N | 99 | 99 |  | 99 | 99 |  |
|  | |  |  |  |  |  |  |
| Rocking backwards | |  |  |  | |  |  |
|  | Negative | 23 | 23 |  | 23 | 52 |  |
|  | Positive | 34 | 34 | 1.00 (N/A) | 34 | 29 | 0.27 (0.13–0.41) |
|  | Undecided | 42 | 42 |  | 40 | 16 |  |
|  | Total N | 99 | 99 |  | 97 | 97 |  |
|  | |  |  |  |  |  |  |
| One-leg stance | |  |  |  | |  |  |
|  | Negative | 44 | 46 |  | 44 | 75 |  |
|  | Positive | 43 | 41 | 0.97 (0.92–1.01) | 43 | 18 | 0.21 (0.03–0.38) |
|  | Undecided | 12 | 12 |  | 12 | 6 |  |
|  | Total N | 99 | 99 |  | 99 | 99 |  |
|  | |  |  |  |  |  |  |
| Prone knee bend | |  |  |  | |  |  |
|  | Negative | 55 | 55 |  | 54 | 65 |  |
|  | Positive | 40 | 40 | 0.96 (0.91–1.01) | 39 | 31 | 0.62 (0.47–0.78) |
|  | Undecided | 4 | 4 |  | 3 | 0 |  |
|  | Total N | 99 | 99 |  | 96 | 96 |  |
|  | |  |  |  |  |  |  |
| Posterior pelvic tilt (PPT) | |  |  |  | |  |  |
|  | Negative | 21 | 21 |  | 21 | 18 |  |
|  | Positive | 77 | 77 | 1.00 (N/A) | 77 | 79 | 0.49 (0.26–0.71) |
|  | Undecided | 1 | 1 |  | 1 | 2 |  |
|  | Total N | 99 | 99 |  | 99 | 99 |  |
|  |  |  |  |  |  |  |  |
| All MCI tests | |  |  |  | |  |  |
|  | Negative | 195 | 194 |  | 194 | 299 |  |
|  | Positive | 303 | 301 | 0.99 (0.98–0.99) | 302 | 229 | 0.63 (0.60–0.66) |
|  | Undecided | 96 | 99 |  | 93 | 61 |  |
|  | Total N | 594 | 594 |  | 589 | 589 |  |
|  |  |  |  |  |  |  |  |
| All MCI tests without PPT^*^ | |  |  |  | |  |  |
|  | Negative | 174 | 173 |  | 173 | 281 |  |
|  | Positive | 226 | 224 | 0.98 (0.97–1.00) | 225 | 150 | 0.59 (0.54–0.64) |
|  | Undecided | 95 | 98 |  | 92 | 59 |  |
|  | Total N | 495 | 495 |  | 490 | 490 |  |
| ^*^All movement control (MCI) tests without posterior pelvic tilt (PPT).  ^**^Kappa value and 95% confidence interval (CI, in brackets).  N/A, not applicable | | | | | | | |
