# Supplementary material for: Association between low back movement control impairment test results and low back pain – Northern Finland birth cohort study 1966
Source: BMC Musculoskelet Disord. 2026 Jul 11;27:842. doi: 10.1186/s12891-026-10176-6 (PMC13647630; doi:10.1186/s12891-026-10176-6)
Supplement: Supplementary file 3 [file 12891_2026_10176_MOESM3_ESM.docx]

| **Supplementary file 3**. Characteristics of study population by movement control impairment (MCI) test category and pain group. | | | | | | | | | | | | | | | | | | | | | | |
| --- | --- | --- | --- | --- | --- | --- | --- | --- | --- | --- | --- | --- | --- | --- | --- | --- | --- | --- | --- | --- | --- | --- |
|  | | | | | | | LBP | | | | Pain group difference | Sciatica | | | | Pain group difference | Sciatica prolonged | | | | | Pain group difference |
|  | | | | | | | Mild | Moderate | | Severe | (p-value^••^) | Mild | Moderate | | Severe | (p-value) | Mild | Moderate | Severe | | | (p-value) |
| Flexion | | | | | | |  | | | |  |  | | | |  |  | | | | |  |
|  | 0 positive test results | | | | | | 43 | 34 | | 33 | 0.343 | 105 | 99 | | 88 | 0.014 | 131 | 2 | 7 | | | 0.346 |
|  | 1 positive test result | | | | | | 95 | 56 | | 53 |  | 161 | 37 | | 15 |  | 217 | 6 | 12 | | |  |
|  | 2 positive test results | | | | | | 68 | 64 | | 59 |  | 153 | 27 | | 15 |  | 204 | 6 | 3 | | |  |
|  | 3 positive test results | | | | | | 38 | 39 | | 30 |  | 81 | 20 | | 27 |  | 120 | 3 | 8 | | |  |
|  |  | | | | | |  |  | |  |  |  |  | |  |  |  |  |  | | |  |
|  | | Total N | | | | | 612 | | | |  | 687 | | | |  | 719 | | | | |  |
|  |  | | | | | |  |  | |  |  |  | | | |  |  | | | | |  |
|  | | | | Missing data^•^ | | | 1295 | | | |  | 1220 | | | |  | 1188 | | | | |  |
|  | | | | | | |  | | | |  |  | | | |  |  | | | | |  |
| Rotation (left) | | | | | | |  | | | |  |  | | | |  |  | | | | |  |
|  | 0 positive test results | | | | | | 25 | 14 | | 11 | 0.329 | 40 | 10 | | 4 | 0.055 | 48 | 2 | 3 | | | 0.839 |
|  | 1 positive test result | | | | | | 88 | 54 | | 66 |  | 192 | 27 | | 34 |  | 242 | 8 | 10 | | |  |
|  | 2 positive test results | | | | | | 141 | 118 | | 104 |  | 282 | 69 | | 50 |  | 392 | 12 | 19 | | |  |
|  | 3 positive test results | | | | | | 77 | 72 | | 69 |  | 163 | 36 | | 45 |  | 229 | 8 | 17 | | |  |
|  |  | | | | | |  |  | |  |  |  |  | |  |  |  |  |  | | |  |
|  | | Total N | | | | | 839 | | | |  | 952 | | | |  | 990 | | | | |  |
|  |  | | | | | |  | | | |  |  | | | |  |  | | | | |  |
|  | | | | Missing data^•^ | | | 1068 | | | |  | 955 | | | |  | 917 | | | | |  |
|  | | | | | | |  | | | |  |  | | | |  |  | | | | |  |
| Rotation (right) | | | | | | |  | | | |  |  | | | |  |  | | | | |  |
|  | 0 positive test results | | | | | | 14 | 10 | | 9 | 0.290 | 31 | 5 | | 7 | 0.977 | 35 | 2 | 4 | | | 0.718 |
|  | 1 positive test result | | | | | | 82 | 59 | | 51 |  | 167 | 36 | | 30 |  | 221 | 10 | 11 | | |  |
|  | 2 positive test results | | | | | | 157 | 113 | | 114 |  | 302 | 63 | | 65 |  | 404 | 14 | 22 | | |  |
|  | 3 positive test results | | | | | | 77 | 85 | | 79 |  | 181 | 39 | | 40 |  | 262 | 7 | 13 | | |  |
|  |  | | | | | |  |  | |  |  |  |  | |  |  |  |  |  | | |  |
|  | | Total N | | | | | 850 | | | |  | 966 | | | |  | 1005 | | | | |  |
|  |  | | | | | |  | | | |  |  | | | |  |  | | | | |  |
|  | | | | Missing data^•^ | | | 1057 | | | |  | 941 | | | |  | 902 | | | | |  |
|  | | | | | | |  | | | |  |  | | | |  |  | | | | |  |
| Rotation (both) | | | | | | |  | | | |  |  | | | |  |  | | | | |  |
|  | 0 positive test results | | | | | | 32 | 22 | | 13 | 0.403 | 60 | 10 | | 8 | 0.514 | 69 | 3 | 5 | | | 0.868 |
|  | 1 positive test result | | | | | | 93 | 65 | | 72 |  | 195 | 43 | | 34 |  | 261 | 9 | 10 | | |  |
|  | 2 positive test results | | | | | | 130 | 108 | | 97 |  | 267 | 60 | | 51 |  | 362 | 13 | 19 | | |  |
|  | 3 positive test results | | | | | | 61 | 58 | | 56 |  | 130 | 24 | | 34 |  | 189 | 4 | 10 | | |  |
|  |  | | | | | |  |  | |  |  |  |  | |  |  |  |  |  | | |  |
|  | | Total N | | | | | 807 | | | |  | 916 | | | |  | 954 | | | | |  |
|  |  | | | | | |  |  | |  |  |  | | | |  |  | | | | |  |
|  | | | | Missing data^•^ | | | 1100 | | | |  | 991 | | | |  | 953 | | | | |  |
|  | | | | | | |  | | | |  |  | | | |  |  | | | | |  |
| Flexion-rotation (left) | | | | | | |  | | | |  |  | | | |  |  | | | | |  |
|  | 0 positive test results | | | | | | 52 | 34 | | 29 | 0.371 | 96 | 22 | | 11 | 0.060 | 122 | 6 | 5 | | | 0.760 |
|  | 1 positive test result | | | | | | 170 | 126 | | 139 |  | 364 | 66 | | 67 |  | 478 | 13 | 26 | | |  |
|  | 2 positive test results | | | | | | 112 | 101 | | 86 |  | 222 | 56 | | 58 |  | 321 | 11 | 18 | | |  |
|  |  | | | | | |  |  | |  |  |  |  | |  |  |  |  |  | | |  |
|  | | Total N | | | | | 849 | | | |  | 962 | | | |  | 1000 | | | | |  |
|  |  | | | | | |  |  | |  |  |  | | | |  | | | | | |  |
|  | | | | | Missing data^•^ | | 1058 | | | |  | 945 | | | |  | 907 | | | | |  |
|  |  | | | | | |  | | | |  |  | | | |  |  | | | | |  |
| Flexion-rotation (right) | | | | | | |  | | | |  |  | | | |  |  | | | | |  |
|  | 0 positive test results | | | | | | 48 | 32 | | 30 | 0.624 | 95 | 22 | | 15 | 0.489 | 122 | 9 | 6 | | | 0.172 |
|  | 1 positive test result | | | | | | 174 | 133 | | 133 |  | 358 | 66 | | 79 |  | 468 | 13 | 28 | | |  |
|  | 2 positive test results | | | | | | 113 | 106 | | 96 |  | 237 | 57 | | 52 |  | 345 | 11 | 16 | | |  |
|  |  | | | | | |  |  | |  |  |  |  | |  |  |  |  |  | | |  |
|  | | Total N | | | | | 865 | | | |  | 980 | | | |  | 1018 | | | | |  |
|  |  | | | | | |  | | | |  |  | | | |  |  | | | | |  |
|  | | | | | Missing data^•^ | | 1042 | | | |  | 927 | | | |  | 889 | | | | |  |
|  |  | | | | | |  | | | |  |  | | | |  |  | | | | |  |
| Flexion-rotation (both) | | | | | | |  | | | |  |  | | | |  |  | | | | |  |
|  | 0 positive test results | | | | | | 73 | 50 | | 38 | 0.167 | 138 | 29 | | 19 | 0.460 | 177 | 10 | | 7 | 0.121 | |
|  | 1 positive test result | | | | | | 144 | 122 | | 132 |  | 323 | 68 | | 64 |  | 428 | 10 | | 27 |  |  |
|  | 2 positive test results | | | | | | 102 | 84 | | 73 |  | 197 | 42 | | 47 |  | 287 | 9 | | 10 |  |  |
|  |  | | | | | |  |  | |  |  |  |  | |  |  |  |  | |  |  | |
|  | | Total N | | | | | 818 | | | |  | 927 | | | |  | 965 | | | |  | |
|  |  | | | | | |  | | | |  |  | | | |  |  | | | | |  |
|  | | | | | Missing data^•^ | | 1089 | | | |  | 980 | | | |  | 942 | | | | |  |
|  |  | | | | | |  | | | |  |  | | | |  |  | | | | |  |
| Extension-rotation (left) | | | | | | |  | | | |  |  | | | |  |  | | | | |  |
|  | 0 positive test results | | | | | | 63 | | 46 | 44 | 0.971 | 116 | 23 | | 22 | 0.948 | 153 | 5 | 8 | | | 0.702 |
|  | 1 positive test result | | | | | | 205 | | 160 | 147 |  | 414 | 87 | | 85 |  | 567 | 16 | 28 | | |  |
|  | 2 positive test results | | | | | | 262 | | 204 | 203 |  | 508 | 109 | | 115 |  | 704 | 24 | 48 | | |  |
|  |  | | | | | |  | |  |  |  |  |  | |  |  |  |  |  | | |  |
|  | | Total N | | | | | 1334 | | | |  | 1479 | | | |  | 1553 | | | | |  |
|  |  | | | | | |  | |  |  |  |  |  | |  |  |  |  |  | | |  |
|  | | | | | Missing data^•^ | | 573 | | | |  | 428 | | | |  | 354 | | | | |  |
|  | | | | | | |  | | | |  |  | | | |  |  | | | | |  |
| Extension-rotation (right) | | | | | | |  | | | |  |  | | | |  |  | | | | |  |
|  | 0 positive test results | | | | | | 46 | | 41 | 29 | 0.425 | 87 | 26 | | 18 | 0.510 | 121 | 4 | 7 | | | 0.270 |
|  | 1 positive test result | | | | | | 122 | | 155 | 154 |  | 422 | 84 | | 91 |  | 566 | 25 | 39 | | |  |
|  | 2 positive test results | | | | | | 254 | | 204 | 210 |  | 513 | 103 | | 115 |  | 714 | 16 | 40 | | |  |
|  |  | | | | | |  | |  |  |  |  |  | |  |  |  |  |  | | |  |
|  | | Total N | | | | | 1315 | | | |  | 1459 | | | |  | 1532 | | | | |  |
|  |  | | | | | |  | |  |  |  |  |  | |  |  |  | | | | |  |
|  | | | | | Missing data^•^ | | 592 | | | |  | 448 | | | |  | 375 | | | | |  |
|  | | | | | | |  | | | |  |  | | | |  |  | | | | |  |
| Extension-rotation (both) | | | | | | |  | | | |  |  | | | |  |  | | | | |  |
|  | 0 positive test results | | | | | | 108 | | 83 | 73 | 0.953 | 206 | 45 | | 36 | 0.421 | 270 | 45 | 81 | | | 0.204 |
|  | 1 positive test result | | | | | | 234 | | 176 | 176 |  | 460 | 103 | | 99 |  | 638 | 28 | 33 | | |  |
|  | 2 positive test results | | | | | | 176 | | 137 | 136 |  | 345 | 64 | | 83 |  | 478 | 10 | 32 | | |  |
|  |  | | | | | |  | |  |  |  |  |  | |  |  |  |  |  | | |  |
|  | | Total N | | | | | 1299 | | | |  | 1441 | | | |  | 1512 | | | | |  |
|  |  | | | | | |  | |  |  |  |  |  | |  |  |  | | | | |  |
|  | | | | | | Missing data^•^ | 608 | | | |  | 466 | | | |  | 395 | | | | |  |
|  | | | | | | |  | | | |  |  | | | |  |  | | | | |  |
| Extension | | | | | | |  | | | |  |  | | | |  |  | | | | |  |
|  | 0 positive test results | | | | | | 238 | | 179 | 165 | 0.667 | 467 | 93 | | 95 | 0.845 | 620 | 20 | 37 | | | 0.980 |
|  | 1 positive test result | | | | | | 335 | | 268 | 261 |  | 662 | 142 | | 142 |  | 921 | 28 | 55 | | |  |
|  |  | | | | | |  | |  |  |  |  |  | |  |  |  |  |  | | |  |
|  | | | Total N | | | | 1446 | | | |  | 1601 | | | |  | 1681 | | | | |  |
|  |  | | | | | |  | |  |  |  |  |  | |  |  |  | | | | |  |
|  | | | | | | Missing data^•^ | 461 | | | |  | 306 | | | |  | 226 | | | | |  |
|  | | | | | | |  | | | |  |  | | | |  |  | | | | |  |
| All tests | | | | | | |  | | | |  |  | | | |  |  | | | | |  |
|  | 0 positive test results | | | | | | 8 | | 5 | 5 | 0.137 | 18 | | 4 | 2 | 0.640 | 21 | 1 | 2 | | | 0.789 |
|  | 1–2 positive test results | | | | | | 109 | | 61 | 61 |  | 199 | | 39 | 29 |  | 261 | 7 | 10 | | |  |
|  | 3–5 positive test results | | | | | | 112 | | 106 | 90 |  | 242 | | 47 | 50 |  | 337 | 9 | 13 | | |  |
|  |  | | | | | |  | |  |  |  |  | |  |  |  |  |  |  | | |  |
|  | | | Total N | | | | 557 | | | |  | 630 | | | |  | 661 | | | | |  |
|  |  | | | | | |  | |  |  |  |  | |  |  |  |  | | | | |  |
|  | | | | | | Missing data^•^ | 1350 | | | |  | 1277 | | | |  | 1246 | | | | |  |
|  | | | | | | | | | | | | | | | | | | | | | | |
| ^•^Missing data are compared to study population (MCI population, n=1907)  ^••^Pain group differences in positive MCI tests were analysed using the Chi-square test. | | | | | | | | | | | | | | | | | | | | | | |
